# Supplementary material for: Comparative evaluation of iStent versus iStent inject W combined with phacoemulsification in open angle glaucoma
Source: PLoS One. 2024 Feb 5;19(2):e0297514. doi: 10.1371/journal.pone.0297514 (PMC10843132; doi:10.1371/journal.pone.0297514)
Supplement: Attachment — Submitted filename: Response_to_Reviewers.docx [file pone.0297514.s002.docx]

**Response to Reviewers**

**Response to the Editor**

1. Please ensure that your manuscript meets PLOS ONE's style requirements, including those for file naming. The PLOS ONE style templates can be found at 
<https://journals.plos.org/plosone/s/file?id=wjVg/PLOSOne_formatting_sample_main_body.pdf> and 
<https://journals.plos.org/plosone/s/file?id=ba62/PLOSOne_formatting_sample_title_authors_affiliations.pdf>

Response: Thank you for your suggestion. We have ensured that the manuscript meets PLOS ONE's style requirements and have referred to the journal’s style templates.

2. In the ethics statement in the Methods, you have specified that verbal consent was obtained. Please provide additional details regarding how this consent was documented and witnessed, and state whether this was approved by the IRB.

Response: Thank you for your valuable comment. We have added the necessary IRB-approval permit number in the manuscript.

3. We note that Figures 1.1, 1.2, 2.1 and 2.2 in your submission contain copyrighted images. All PLOS content is published under the Creative Commons Attribution License (CC BY 4.0), which means that the manuscript, images, and Supporting Information files will be freely available online, and any third party is permitted to access, download, copy, distribute, and use these materials in any way, even commercially, with proper attribution. For more information, see our copyright guidelines: <http://journals.plos.org/plosone/s/licenses-and-copyright>.

We require you to either (1) present written permission from the copyright holder to publish these figures specifically under the CC BY 4.0 license, or (2) remove the figures from your submission:
 a. You may seek permission from the original copyright holder of Figures 1.1, 1.2, 2.1 and 2.2 to publish the content specifically under the CC BY 4.0 license. 
 
We recommend that you contact the original copyright holder with the Content Permission Form (<http://journals.plos.org/plosone/s/file?id=7c09/content-permission-form.pdf>) and the following text:
“I request permission for the open-access journal PLOS ONE to publish XXX under the Creative Commons Attribution License (CCAL) CC BY 4.0 (<http://creativecommons.org/licenses/by/4.0/>). Please be aware that this license allows unrestricted use and distribution, even commercially, by third parties. Please reply and provide explicit written permission to publish XXX under a CC BY license and complete the attached form.”
 
Please upload the completed Content Permission Form or other proof of granted permissions as an "Other" file with your submission. 
 
In the figure caption of the copyrighted figure, please include the following text: “Reprinted from [ref] under a CC BY license, with permission from [name of publisher], original copyright [original copyright year].”
 
b. If you are unable to obtain permission from the original copyright holder to publish these figures under the CC BY 4.0 license or if the copyright holder’s requirements are incompatible with the CC BY 4.0 license, please either i) remove the figure or ii) supply a replacement figure that complies with the CC BY 4.0 license. Please check copyright information on all replacement figures and update the figure caption with source information. If applicable, please specify in the figure caption text when a figure is similar but not identical to the original image and is therefore for illustrative purposes only.

Response: Thank you for your suggestion. As we were unable to obtain the copyright permissions, we have removed the figures 1.1, 1.2, 2.1 and 2.2 from the revised manuscript, as per your suggestion.

**Reviewer 1 comments**

1. Introduction: I would remove the discussion regarding the comparison between the design of each device to make the introduction more concise. If wanting to include in the paper I would add more detail to the subheading regarding surgical technique and surgical device

Response: We have deleted the discussion regarding the comparison between the design of each device to make the introduction more concise.

2. Discussion: I think the discussion can be made considerably more concise. Instead of simply restating the results in an organized fashion, I would try and summarize efficacy into one paragraph, safety into another and explore factors for failure in the third to make it better for readers of the manuscript.

Response: We have reorganized the Discussion section to concisely describe the efficacy, safety, and factors for failure.

3. Results: I would consider stratifying results by baseline IOP. For good example see citation [1]. This would be helpful to understand how a patient with baseline IOP ranging from 18-20 did and see the overall breakdown of baseline IOP.

Ferguson TJ, Swan RJ, Bleeker A, Dockter Z, Karpuk KL, Schweitzer J, Ibach M, Berdahl JP. Trabecular microbypass stent implantation in pseudoexfoliative glaucoma: long-term results. Journal of Cataract & Refractive Surgery. 2020 Sep 1;46(9):1284-9.

Response: We had already attempted to stratify the baseline IOP of 18–20 mmHg and study them separately, but the small number of n (eyes) to stratify prevented meaningful statistical analysis.

Reviewer 2 Comments

1. As described in the title “comparative evaluation of iStent versus iStent inject W…”, the main outcome in this study is the surgical outcome between iStent versus iStent inject W. The result are no statistically significant differences between the two procedures. However, the sample size calculation does not seem to be determined before the study.
Response: No sample size calculations were done in advance for this study. Therefore, as you have indicated, the power of this study may have been inadequate. We would like to add that as a limitation.

Minor points

1. In criteria of selection and patient visitation of the material and methods, the authors described “post-operative BCVA and VF were also compared to the corresponding pre-operative values on day 1, week 1, months 1, and 3, and at the final visit after surgery.” However, there are no data on each follow-up visits.

Response: For visual acuity, Table 4 shows the results at the time of the method description. Regarding VF, we have corrected the method, as it was not measured at the time points described.

2. Figures 1 and 2 should be deleted from the manuscript. The readers can find the pictures by internet because they were provided by the manufacturer “Glaukos.”

Response: We have removed the figures from the Manuscript.

The authors did not find the statistical difference of the efficacy between iStent vs iStent Inject. How many sample size do the authors estimate to detect the statistical significance? Sample size calculation is not described in the statistical analysis of the material and methods.

Response: No sample size calculations were performed in advance for this study. Therefore, as you have indicated, the power may have been inadequate. We would like to add that as a limitation.

3. In Tables, legends are described. Legend sentences are removed from the tables.
Response: I would like to inform you that the tables are correctly formatted as required by the journal submission guidelines. Legends are provided below the table as required.

4. In factors affecting cumulative survival in the results, preoperative IOP was a significant factor. Table 7 indicates that lower IOP is a significant factor for surgical failure. However, there are no descriptions in the result. In univariate analysis, operator is a significant factor. Who is a significant factor for surgical failure?

Response: Although there seems to be a difference in the outcomes between surgeons, the results are based on univariate analysis. Since confounding factors may be involved, a multivariate analysis was performed, and operator was not a significant factor influencing the results. In other words, we do not believe that the difference in performance between surgeons in this study is significant.

5. In Table 7, there are no units. Therefore, it is difficult to understand the values.

Response: The table shows Hazard Ratios and Confidence intervals, and thus there are no units.

6. In discussion, although the authors describe “this study is the first report to compare the post-operative results of iStent and inject W.” However, there are several retrospective reports about iStent vs iStent inject. iStent inject W is a minor revision of iStent inject. Therefore, the efficacy of iStent inject W seems to be the same as iStent inject.

Response: We have added a sentence at the beginning of the Discussion to acknowledge the presence of several retrospective studies.

7. The manuscript format does not seem to be designed well. Please edit the manuscript, tables and figures again.

Response: I have checked the submission guidelines again and ensured full compliance with the formatting requirements.

Editor’s Comments

We've checked your submission and before we can proceed, we need you to address the following issues:

1. We notice that your manuscript file was uploaded on February 6, 2023. Please can you upload the latest version of your revised manuscript as the main article file, ensuring that does not contain any tracked changes or highlighting.  This will be used in the production process if your manuscript is accepted. Please follow this link for more information: [http://blogs.PLOS.org/everyone/2011/05/10/how-to-submit-your-revised-manuscript/](http://blogs.plos.org/everyone/2011/05/10/how-to-submit-your-revised-manuscript/)
Response: We have uploaded the correct file.

2. Please upload a Response to Reviewers letter which should include a point by point response to each of the points made by the Editor and / or Reviewers. (This should be uploaded as a 'Response to Reviewers' file type.) Please follow this link for more information: [http://blogs.PLOS.org/everyone/2011/05/10/how-to-submit-your-revised-manuscript/](http://blogs.plos.org/everyone/2011/05/10/how-to-submit-your-revised-manuscript/)

Response: We have uploaded a Response to Reviewer letter containing our responses to the reviewers’ and editor’s comments.

3. Please amend the title either on the online submission form or in your so that they are identical.
Response: We have revised the title and resubmitted the manuscript.

4. Please upload a copy of Figures 2 and 3 which you refer to in your text on pages 5 and 17. Or if the figure is no longer to be included as part of the submission please remove all reference to it within the text.
Response: We have corrected the figures for consistency between what we quote in the text and the figure uploaded.
