# Supplementary material for: Comparative evaluation of iStent versus iStent inject W combined with phacoemulsification in open angle glaucoma
Source: PLoS One. 2024 Feb 5;19(2):e0297514. doi: 10.1371/journal.pone.0297514 (PMC10843132; doi:10.1371/journal.pone.0297514)
Supplement: Attachment — Submitted filename: Abhinav Golla_istent Review.docx [file pone.0297514.s003.docx]

Abhinav Golla

Comparison of iStent and iStent inject W

1. Introduction, line 63-65: “Recently, minimally invasive glaucoma surgeries (MIGS) have emerged, and they are expected to reduce IOP more safely and effectively.”

When compared to trabeculectomies or tube shunts, MIGS carry a better safety profile, however, it is not clear that they are more effective, so you could consider deleting the word “effectively”.

2. Introduction

A suggestion that I wanted to bring up, though not critical- Is the iStent still available for use, or is only the iStent inject W sold? I realize that in a previous revisions of your manuscript, the diagram of the iStent and iStent inject W with measurements of the lumen sizes may have been removed, which is okay. The differences between the iStent and iStent inject W are discussed in the discussion section (lines 345-352). However, I believe that readers may want to know why the two types of stents are being compared in the first place, especially if they have a choice between the two types of stents. For example, is it simply whether one stent is better than two? Or is it the stent itself. Therefore, it might be helpful to consider describing the differences between the two in the introduction section. Additionally, maybe a quantification of differences in parameters hypothesized to affect pressure and flow, such as difference in lumen size in microns between the two (inject W with 80 micron central inlet and 80 micron outlet with four 50 micron side outlets and 360 micron length) could be included.

3. “Criteria of selection and patient visitation”, line 109

I would recommend changing the “visitation” wording to “patient visits” or “Inclusion and Exclusion Criteria” for example. Visitation has multiple meanings.

4. Line 116-118, “In addition, all patients who underwent cataract surgery with either iStent or inject W insertion and those who could not be inserted iStent or two inject W were excluded from the analysis.”

Wording could be clarified to say that for patients in whom the iStent or iStent inject W was attempted but unsuccessfully inserted were excluded from the study.

5. In discussion of complications- Given the number of total patients and the overall rarity of complications, it is difficult to comment whether or not there is any difference. For example, coagula in anterior chamber and “Other” (iritis, macular edema, etc.) does have a large magnitude difference between the two groups, though not statistically significant.

6. Discussion, line 293-294. Typo: “But,this However, this study is the first report to compare the post-operative results of iStent and inject W.”

“But,this” should be deleted.

7. Discussion, 295. “mid-term” results.

This should be quantified as an exact month or year, instead of mid-term (ambiguous length of time).

8. Limitations – Line 394-395.

Just a comment - the discussion that no minimum clock hour distance between the two stents for the inject W was set is important, as there is some evidence of differential and segmental flow of aqueous out of Schlemm’s Canal. This means that stents placed further apart may *potentially* have a higher probabilistic chance of accessing high flow regions rather than simply providing more surface area of outflow. Alternatively, maybe placing two stents side by side in an area of good flow provides even better pressure reduction, would in a large group of patients cancel out the effect of having higher probabilistic chance of accessing high flow areas.

9. Discussion, line 369-371: “On the contrary, the eyes with low baseline IOP have a lower pressure gradient, which does not allow significant aqueous outflow through the stent.”

This is a very interesting point regarding outflow and percent reduction of IOP, as people with lower IOPs may potentially be “closer” to their episceral venous pressure “floor” and thus have a reduced ability to lower pressure effectively by methods that do not bypass the EVP, as you pointed out.

I enjoyed reading the paper and I believe that it addresses the question you all sought out to answer.
