# Supplementary material for: Comparative evaluation of iStent versus iStent inject W combined with phacoemulsification in open angle glaucoma
Source: PLoS One. 2024 Feb 5;19(2):e0297514. doi: 10.1371/journal.pone.0297514 (PMC10843132; doi:10.1371/journal.pone.0297514)
Supplement: Attachment — Submitted filename: Response_to_Reviewers.docx [file pone.0297514.s004.docx]

Review Comments to the Author

Reviewer #2: Although the reviewer commented the sample size calculation for the comparison of the efficiencies between the two types of iStent. But, the authors have not shown it. The authors do not seem to be able to conclude the difference of the efficiencies between them.

Answer: Thank you for your comment. We appreciate your insights. In this study, we did not conduct a pre-study sample size calculation, which may have been responsible for the lower statistical power. Therefore, we have addressed this limitation on Lines 432-4 of the manuscript.

フォームの始まり

In discussion: The authors described “this study is the first report to compare the post-operative results of iStent and inject W.” But, a similar study has already been reported by other authors “Kanda S, Fujishiro T, Karakawa A, Nakagawa S, Ishii K. Clinical Outcomes of Phacoemulsification in Japanese Patients Receiving First and Revised Second-Generation Trabecular Microbypass Stents. Asia Pac J Ophthalmol (Phila). 2023 May-Jun 01;12(3):279-283. doi: 10.1097/APO.0000000000000611. Epub 2023 May 15. PMID: 37494255.”

Answer: When we initially submitted our report, there were no similar studies published in the literature. However, as of now, one such study is available. Therefore, we have revised the statement from "this study is the first report to compare the post-operative results of iStent and inject W" to “However, our study is valuable owing to the scarcity of studies comparing the outcomes of iStent and inject W [15]."

フォームの始まり

Reviewer #3: Please see attached document, also pasted here:

1. Introduction, line 63-65: “Recently, minimally invasive glaucoma surgeries (MIGS) have emerged, and they are expected to reduce IOP more safely and effectively.”

When compared to trabeculectomies or tube shunts, MIGS carry a better safety profile, however, it is not clear that they are more effective, so you could consider deleting the word “effectively”.

Answer: Thank you for your comments. Due to the potential for misunderstanding, we will omit the term "effectively" from the sentence in question.

2. Introduction

A suggestion that I wanted to bring up, though not critical- Is the iStent still available for use, or is only the iStent inject W sold? I realize that in a previous revisions of your manuscript, the diagram of the iStent and iStent inject W with measurements of the lumen sizes may have been removed, which is okay. The differences between the iStent and iStent inject W are discussed in the discussion section (lines 345-352). However, I believe that readers may want to know why the two types of stents are being compared in the first place, especially if they have a choice between the two types of stents. For example, is it simply whether one stent is better than two? Or is it the stent itself. Therefore, it might be helpful to consider describing the differences between the two in the introduction section. Additionally, maybe a quantification of differences in parameters hypothesized to affect pressure and flow, such as difference in lumen size in microns between the two (inject W with 80 micron central inlet and 80 micron outlet with four 50 micron side outlets and 360 micron length) could be included.

Answer: Thank you for your feedback. Both iStent and iStent inject W are currently available in Japan. A previous reviewer suggested that the figures related to iStent and inject W should be removed, and that the textual discussion on structural differences is unnecessary. Consequently, these sections have been deleted from the revised version. As you pointed out, we believe that the potential for improved outcomes lies in the simple increase in outflow due to the straightforward insertion of one or two stents and refinement of the device's shape for more direct access to Schlemm's canal.

フォームの始まり

3. “Criteria of selection and patient visitation”, line 109

I would recommend changing the “visitation” wording to “patient visits” or “Inclusion and Exclusion Criteria” for example. Visitation has multiple meanings.

Answer: The subsection heading has been modified based on your suggestion.

4. Line 116-118, “In addition, all patients who underwent cataract surgery with either iStent or inject W insertion and those who could not be inserted iStent or two inject W were excluded from the analysis.”

Wording could be clarified to say that for patients in whom the iStent or iStent inject W was attempted but unsuccessfully inserted were excluded from the study.

Answer: I have made the following revisions as suggested.

“In addition, patients in whom the iStent or iStent inject W was attempted but unsuccessfully inserted were excluded from the study.”

5. In discussion of complications- Given the number of total patients and the overall rarity of complications, it is difficult to comment whether or not there is any difference. For example, coagula in anterior chamber and “Other” (iritis, macular edema, etc.) does have a large magnitude difference between the two groups, though not statistically significant.

Answer: Thank you for your suggestion, we have noted this point in the Limitation as follows (Lines 393-6).

“The relatively low incidence of complications compared to the overall number of patients could have obfuscated accurate assessment of the differences between the two groups. While it is evident that the procedure is relatively safe, a more precise evaluation of significant differences in complications between the two groups would require a larger cohort.”

6. Discussion, line 293-294. Typo: “But,this However, this study is the first report to compare the post-operative results of iStent and inject W.”

“But,this” should be deleted.

Answer: We have deleted the erroneous phrase.

7. Discussion, 295. “mid-term” results.

This should be quantified as an exact month or year, instead of mid-term (ambiguous length of time).

Answer: Thank you very much for this insightful comment. We have provided specific numerical values, as requested.

8. Limitations – Line 394-395.

Just a comment - the discussion that no minimum clock hour distance between the two stents for the inject W was set is important, as there is some evidence of differential and segmental flow of aqueous out of Schlemm’s Canal. This means that stents placed further apart may potentially have a higher probabilistic chance of accessing high flow regions rather than simply providing more surface area of outflow. Alternatively, maybe placing two stents side by side in an area of good flow provides even better pressure reduction, would in a large group of patients cancel out the effect of having higher probabilistic chance of accessing high flow areas.

Answer: Thank you for this insightful comment. I believe you are correct. Currently, we are investigating whether the outcomes differ based on the distance between the placement of two iStent inject W devices in our facility. We hope to report our findings once they are compiled.

9. Discussion, line 369-371: “On the contrary, the eyes with low baseline IOP have a lower pressure gradient, which does not allow significant aqueous outflow through the stent.”

This is a very interesting point regarding outflow and percent reduction of IOP, as people with lower IOPs may potentially be “closer” to their episceral venous pressure “floor” and thus have a reduced ability to lower pressure effectively by methods that do not bypass the EVP, as you pointed out.

Answer: Thank you very much for your comments.
